# Application of nanotechnology in food: processing, preservation, packaging and safety assessment

**DOI:** 10.1016/j.heliyon.2022.e11795

**Published:** 2022-11-21

**Authors:** Rahul Biswas, Mahabub Alam, Animesh Sarkar, Md Ismail Haque, Md. Moinul Hasan, Mominul Hoque

**Affiliations:** Department of Food Engineering and Tea Technology, Shahjalal University of Science and Technology, Sylhet 3114, Bangladesh

**Keywords:** Nanotechnology, Processing, Preservation, Packaging, Safety assessment

## Abstract

Even though nanotechnology is extensively applied in agriculture, biochemistry, medicine and many other sectors, it is a developing field that conforms to new and more complex applications in food systems as compared to other technologies. It offers a viable strategy for integrating cutting-edge technology into a wide range of operations related to the production, development, fabrication, packaging, storage and distribution of food. The most fundamentally sophisticated technology in nano-based food science, nanoparticles deal with a wide range of nanostructured materials and nano methods, including nanofood, nanotubes, nanocomposites, nano packaging, nanocapsules, nanosensors, liposomes, nanoemulsions, polymeric nanoparticles and nanoencapsulation. This method is developed to increase food solubility and shelf life, availability of bioactive chemical, the protection of food constituents, nutritional supplementation, fortification and food or constituent delivery. Additionally, it serves as an antibacterial agent by generating reactive oxygen species (ROS) which cause bacterial DNA damage, protein denaturation and cell damage. Although the use of nanotechnology in food applications is advancing, there are certain negative or dangerous effects on health related to the toxicity and dangers of ingesting nanoparticles in food. The use of nanotechnology in the food industry, notably in processing, preservation and packaging, with its promising future, was addressed in this study. The toxicity of nanoparticles in food as well as its development in food safety assessments with certain areas of concern were also reviewed.

## 1 Introduction

Nanotechnology is an interdisciplinary science that integrates several disciplines including biology, chemical, mechanical and electronics engineering to comprehend, manipulate and build devices/systems with remarkable functionalities and qualities at the atomic/molecular/supramolecular levels (de Francisco and García-Estepa, 2018). This technology involves the study of structures, devices and materials through construction, characterization, manufacture and manipulation with a minimum one length dimension of 1–100 nm size, and the resultant material has physicochemical properties that differ considerably from the features of macroscale materials composed of the same ingredient when particle size is reduced below this limit (Chellaram et al., 2014). Nanoparticles are thought to be minuscule objects that behave as a single unit with distinct qualities and performance, resulting in a new degree of engagement (Ozimek et al., 2010). In comparison to larger particles (with the same composition), nanoparticles appear to have better chemical and biochemical action, catalytic behavior, penetrability, enzymatic activation and quantum characteristics due to their larger surface area and mass transfer rates (Avella et al., 2007). Nanomaterials are categorized according to their size, properties and structure. Solubility, bioavailability, diffusivity, optics, color, strength, intoxicate, magnetism and thermodynamics are all desirable physiochemical properties of such nanomaterials with a high surface volume ratio (Sahoo et al., 2021).

**Figure 1:**
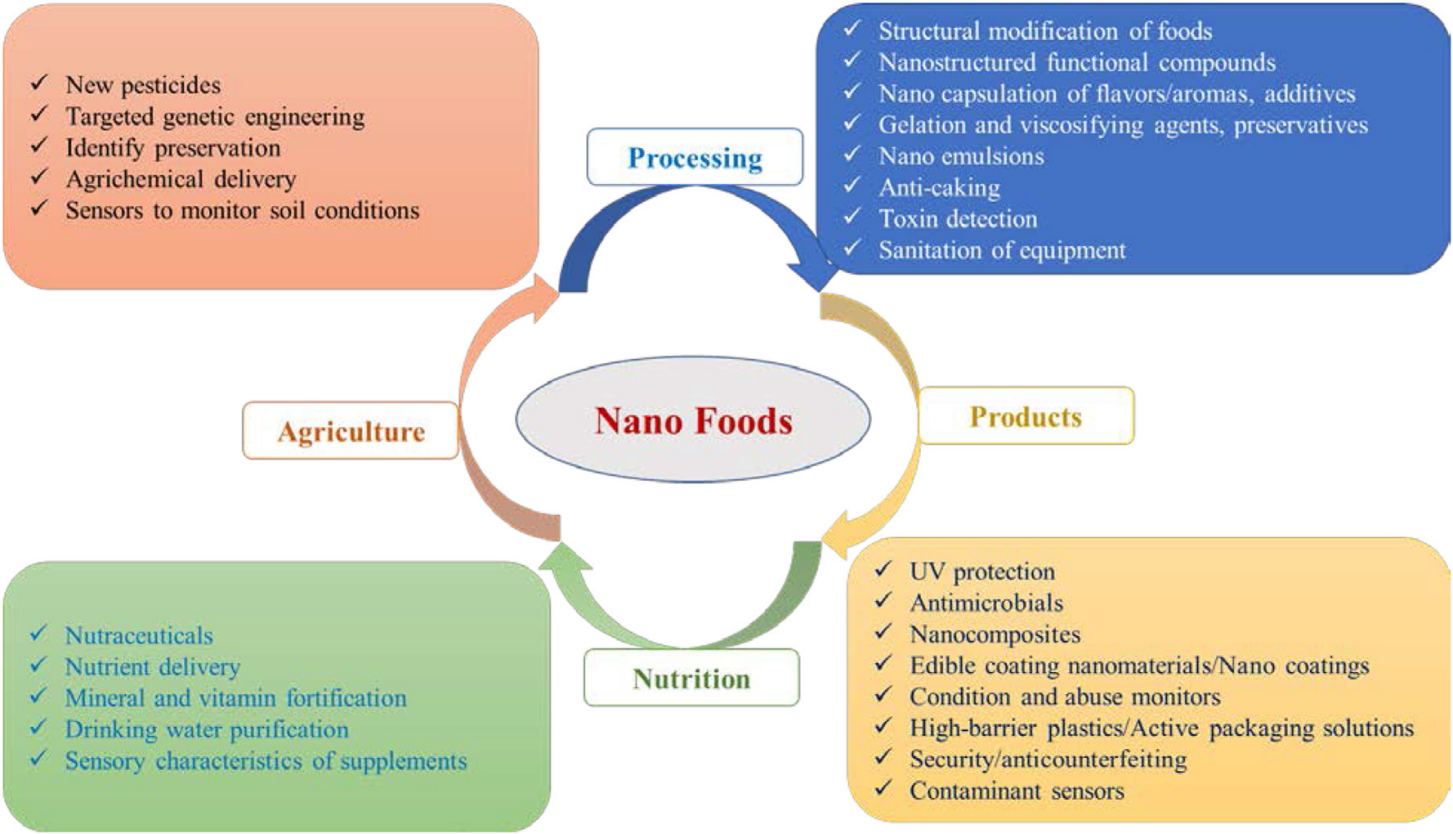
Feasible applications of nanotechnology in all fields including agriculture to food processing safety, packaging, and nutrition of food science (Duncan, 2011; Pathakoti et al., 2017; Sahoo et al., 2021).

Nanotechnology has introduced alternative techniques to food processing in terms of both improving physicochemical qualities and increasing nutrient stability and bioavailability (Patra et al., 2018). Nanoparticles have exceptional mesoscopic features, including larger surface area, high reactivity, tiny particle size, high strength, quantum effects and ductility, which is why they are used across a variety of industries (Ariyarathna et al., 2017; Omerović et al., 2021). The modernization of the food and agriculture industries is roughly identical to that of medication delivery and pharmaceuticals (López-Rubio et al., 2019). Because of their distinctive attributes that differ from their bulk materials, such as physicochemical and biological characteristics, investigations on the synthesizing, categorization, applications and evaluations of nanomaterials have facilitated scientific innovation to improve and modify the whole agri-food sector in new decades (Bouwmeester et al., 2018; Siddiqi et al., 2018). Nanotechnology's application in the food systems has resulted in a large variety of novel products with better food quality attributes such as texture, taste, sensory properties, stability, etc (Anandharamakrishnan and Parthasarathi, 2019). Nanotechnology has already been highlighted as a feasible use in nearly every area of the food sector by scientists and industry groups including agriculture to food processing, safety, packaging and nutrient delivery (illustrated in Figure 1) (Pathakoti et al., 2017; Sahoo et al., 2021). Numerous researches have been led to investigate the feasibility of using nanoparticles in food quality assurance, packaging development, food safety implement and the production of food products with changed function and nutrition (Ariyarathna et al., 2017; Noruzi, 2016; Pathakoti et al., 2017; Singh et al., 2017; Wyser et al., 2016). In addition, nanotechnology involves exposing food-related diseases, designing adequate nutrition regimens for a wide range of target demographics, geriatric population and circumstances as well as ensuring the sustainability of food production through nanoencapsulation. Besides, nutrition nano-therapy can build smart/intelligent systems for regulated nutrient delivery with the development of novel products through food fortification (Sahoo et al., 2021).

Preservatives, flavoring agents, encapsulated food ingredients, antimicrobial sensors, packaging compounds, other nanoparticles and nanoscale food additives are employed to affect the nutritional content and enhance product shelf life, aroma, texture, among other things and they may also be used to locate food pathogens that provide clues about the quality standards of food (Bott et al., 2014). Nanotechnology is used extensively in food preservation, food additives, and food packaging as an antimicrobial compound (particularly Cu/CuO, Ag, MgO, TiO_2_, ZnO, carbon dots, mesoporous particles and graphene etc.) (Omerović et al., 2021; Pathakoti et al., 2017). In comparison to traditional packaging methods, nanotechnology has several advantages, including improved mechanical barrier, heat resistant properties and biodegradability (De Azeredo, 2009). Nanomaterials can be utilized to detect food deterioration using nanosensors due to their increased antibacterial properties (McClements and Xiao, 2012). Antimicrobial packaging (structured polymeric films) or encapsulating materials limit the development phase of microorganisms on the packed food's surface by distributing active substances onto the food or into the external area (Drago et al., 2020). Antimicrobial nanoparticles are used in active packaging to protect food against detrimental and spoilage-causing bacteria to extend shelf-life and quality freshness. They are also included in the active packaging to make it stronger, lighter, and less O_2_ accessible (Hoseinnejad et al., 2018; Omerović et al., 2021; Sharma et al., 2020).

Nanotechnology is utilized in the food sector to enhance food security by employing nanosensors to identify infections or contamination in food throughout manufacturing, processing, packaging, storage and transport (Nile and Kai, 2021; Wang et al., 2021). However, the application of modified nanoparticles in food items certainly raises human oral nanoparticles exposure (Cao et al., 2016). Oral consumption of nanoparticle-enhanced food and ingestion of nanoparticles that have migrated from packaging is a major source of human exposure to nanoparticles (Wang et al., 2021). According to some studies, ingestion of nanoparticles has been linked to protein denaturation (Hong et al., 2017), stimulation of oxidative stress responses (Khanna et al., 2015), DNA damage (Lu et al., 2015), as well as other biological consequences. Oral exposure to nanomaterials, particularly solid nanoparticles, has been linked to gastrointestinal and secondary organ damage in several investigations (Cao et al., 2016). This emphasizes the need of paying close attention to the application of nanoparticles in foodstuff. As a result, determining the degree of exposure and developing efficient techniques to investigate the toxicity and food safety of nanoparticles is a significant pragmatic issue that must be addressed before adequate monitoring of nanoparticles in food can be implemented (Sahoo et al., 2021; Wyser et al., 2016).

In this study, we analyzed and highlighted the pertinent research on nanoparticles used in several food industries, particularly the application and improvement of nanocomposite based active or smart food packaging, with an emphasis on antibacterial nanofillers and biodegradable polymers and explored the toxicity, evaluated the safety assessment relevant to food nanoparticles.

## 2 Food nanostructured materials and their applications

Some foods include nanosized elements that are distinct from synthetically produced nanomaterials. In food, the most important synthetic nanostructured materials are polymeric/biopolymeric nanoparticles (protein), nanoemulsions, liposomes and nanocomposites with several types of nano colloidal forms such as: (i) Nanoparticles (20–200 nm)- generally composed of biodegradable polymers for long-term medication or antioxidant release; (ii) Liposomes (100–400 nm)- tiny, spherical synthetic vesicles made mostly of lipid bilayers; (iii) Micelles (10–100 nm)- self-assembling amphiphilic particles that can encapsulate either lipophilic and lipophobic medicines while being stabilized by surfactants; (iv) Nano capsules (10–1000 nm)- encapsulate significant quantities of medicines and nucleic acids including DNA, microRNA, shRNA and siRNA; (v) Nanoconjugates-polymers that have been covalently bonded with medicinal molecules; (vi) Dendrimers (3–20 nm)- monodisperse macromolecules that may use to encapsulate or covalently conjugate medicines, imaging agents, and targeting moieties (Ojha & Kumar, 2018; Xu et al., 2013). Their utilization in a wide range of food-related nanotechnology applications were represented in Figure 1. During the manufacturing and storage of active compounds, these materials implement bioavailability, increase solubility, enable regulated release and preserve bioactive compounds (Chang and Chen, 2005). Nanostructures are made from food-grade materials utilizing simple layer-by-layer processes and a cost-effective methodology and these nanoparticles have distinct physical, chemical and biological characteristics that involve a comprehension of biological and physical processes in the food chain (Pathakoti et al., 2017). Different forms of nanomaterials can be synthesized utilizing a wide range of methods, such as colloids, particles, thin films, clusters, tubes, wires, rods and powders, etc. As indicated in Table 1, these methods are divided into three basic ways for manufacturing nanomaterials. The approach is based on the materials of interest as well as the form of nanostructures used, including nanowires, quantum dots, nanoplates and nanorods (Kulkarni, 2015a; 2015b; 2015c). Mechanical forces and evaporation are commonly used in the physical technique to synthesize nanomaterials. Physical techniques for the formation of nanomaterials include pulse laser ablation, wire discharge, mechanical/high ball milling, physical vapor deposition with consolidation and mechanical chemical synthesis, etc (Satyanarayana and Reddy, 2018). Nanotechnology is predicted to greatly improve four key focus areas in food application: (i) material development with novel functionality; (ii) processing on a micro-and nanoscale; (iii) development of the product; and (iv) the advancement of food safety and biosecurity procedures and instruments (Moraru et al., 2009). Nanostructured materials offer distinctive features that allow for the advancement of new, strong-performance nanomaterials that will have a significant influence on food processing, preservation, packaging and storage.

**Table 1:** Several types of techniques for synthesis of nanostructured materials (Sahoo et al., 2021).

Physical techniques/methods		Chemical techniques/methods		Biological techniques/methods	
Pulse laser ablation	second harmonic generation Nd:YAG (neodymium-doped yttrium aluminum garnet; Nd:Y_3_Al_5_O_12_) type laser is mostly used	Sono-chemical method	simple, ambient operating conditions, ease in controlling the size of nanoparticles	By micro-organisms	metallic nanoparticles, oxide nanoparticles, sulfide nanoparticles, etc. can be produced using this method
	type of laser, number of pulses, pulsing time and type of solvent affect the produced nanoparticles		earlier proposed for synthesizing iron nanoparticles, but now used for various metal and metal oxides		applications are antibacterial agents, biosensors, reaction rates enhancers, etc.
Mechanical chemical synthesis	mechanical energy enables a chemical reaction to occur	Micro-emulsions	the geometry of aggregated nanoparticles is affected by the position of water oil and surfactant phases	Using plant extracts	Ag, Ag_2_O, TiO_2_, Cu, Au, and CdS nanoparticles, etc. can be produced using this method
	the nanoparticles are recovered through suitable solvent washing		surfactant aggregates can range in size from 1 to 100 nm		applications are antibacterial, catalytic, cytotoxicity, luminescence, etc.
Phase vapor deposition	particle size and their distribution depend upon rate of evaporation and pressure of gases	Electro-chemical method	electricity is the driving or controlling force		
	metal compound can be formed by using reactive gases like H_2_, O_2_, and NH_3_		the method is simple, eco-friendly, low costs, highly flexible, etc.		
High ball milling method	planetary, vibratory, rod, tumbler, etc. are often utilized in nanoparticle synthesis				
	mill type, milling, speed, duration, temperature, size, and size distribution, among other factors, all have an impact on performance				
Pulse wire discharge method	metal, oxide, and nitride nanoparticles are made using this method				
	a high pace of production and a high level of energy efficiency				

Many proteins may transport bioactive molecules and assemble nano complexes (5–100 nm size micelles) due to the uniformity of their fundamental structure and the existence of primary amino groups essential for bioactive element conjugation (Mohammadian et al., 2020). Protein-based nanostructures are categorized in nanotubes, nanogels, nanofibers, nanoparticles, nanofibrils, hollow nanoparticles and so on. Food research and applications are engaged in proteins with nanostructures because they have distinct and specialized functional and biological characteristics. Biocompatibility, biodegradability, amphiphilic nature, foaming, gelation, surface activity, emulsification, film formation and water binding capability are some of these distinguishing features (Sahoo et al., 2021; Verma et al., 2018). For medication delivery and nutritional supplements, nanostructured proteins are effective in interacting with diverse functional groups and bioactive compounds and they give a large surface area for potentially enhanced absorption of active substances, which promotes bioavailability (Fathi et al., 2018; Sahoo et al., 2021). Natural nanostructures may also be found in milk and milk compounds, like casein and whey proteins and different other nanostructured proteins, which have been applied for making several types of bioactive properties like gluten, gelatin, β-lactoglobulin, soy protein, silk fibroin and zein (Zimet and Livney, 2009). Whey protein has also been utilized to preserve bioactive components and encapsulate anthocyanin and folic acid for nutritional supplements (Arroyo-Maya and McClements, 2015).

### 2.1 Food nanostructured materials in processing

Food processing involves different types of numerous unit operations from raw materials to finished products, particularly key processing, preservation, packaging, transportation, distribution and storage where the major purpose is the inactivation of different pathogens/microorganisms and enzymatic activity, removal of toxic substances, nutrients enrichment (fortification-nutrition supplements) and so on activities implementation. As a result, many processes might be considerably improved by using nanotechnology-based applications. Based on process technology, packaging, antimicrobials and food components are some of the potential usages of nanotechnology in foodstuffs that may be characterized as either "direct" or "indirect". Direct usages include the direct integration of nanostructured materials into the food matrix, as well as the indication of their presence and another usage also involving including preservatives, aromas, antioxidants, coloring agents and bioactive constituents like polyphenols, omega-3 fatty acids, vitamins and various types of food components. Indirect usages include the nanostructured particles using smart packaging techniques (McClements and Xiao, 2012), for the hydration of lipids, or the employment of expertly nanostructured catalyzers (Moraru et al., 2009). Nanotechnology advances in food processing are primarily focused on improving food texture, encapsulating food additives or ingredients, generating novel tastes and sensations, regulating flavor release and enhancing the bioavailability of nutrient content (Abbas et al., 2009). Nanotechnology is used to innovate and improve foodstuffs and commodities throughout food processing and production, applying many types of nano techniques and their applications described in Table 2. Antioxidants, antimicrobials, vitamins, flavouring agents, coloring agents and preservatives are among many functional elements utilized in the food industry. These materials are available in a variety of physical and molecular forms (physical states and molecular weights & polarities) (Chau et al., 2007).

**Table 2:** Application of several types of nano techniques for food processing and production in certain fields of various unit operations.

Nano-structured materials/particles	Techniques	Activity	Applications in Food Technology	Reference
Starch, carrageenan, pectin, alginate, guar gum, and xanthan gum are examples of carbohydrates and proteins (vegetable protein, milk, and egg)	Nano emulsions	assist in enhancing the texture and consistency of ice creams	food processing (usages in homogenization, emulsification, capsulation, additivities, nutritional supplementation, fortification, food components preservation, and various types of unit operations), development and production	(Mohammed et al., 2020)
Dammar gum, ester gum, and sucrose-acetate iso-butyrate and some of other ingredients like brominated vegetable oil		facilitate in the distribution and availability of nutrients in food; supports in as a ​balancing agent; creaming and sedimentation are decreased by using this technique		(Liu et al., 2019)
Nano emulsions		produce more food for customized drinks, salad dressings, sweeteners, flavored oils, and other processed foods		(Pathakoti et al., 2017)
Liposomes composed of nanoparticles zein fibers loaded with gallic acid as a nanoliposomes	Nano encapsulation	fertilizers, insecticides, and vaccinations are delivered to the plants; vitamins, additives, enzymes, and other nutrients are delivered through food		(Thakur et al., 2022)
Colloidosomes		boosting the food's nutritional quality; vitamins and minerals are delivered ​in food		(Boonlao et al., 2022)
Nano capsules		bioavailability and effectiveness have been improved; improved oxidation resistance, stability, and preservation of volatile components; fragrance and unpleasant ingredients are trapped in the food; controlled release induced by pH; controlled release induced by taste and moisture		
Nano cuticles (slim shake chocolate & nano tea)		used to nano encapsulate nanoclusters that assist improve the aroma of the shake without adding sugar		(Subhashree & Kumar, 2022)
Daily Boost		used to nano encapsulate enriched vitamins or biologically active ​ingredients in drinks		(Mohammadi et al., 2022)
Color emulsion		beta-carotene, apo carotene, and paprika nano emulsions are all made with this technique		(Atencio et al., 2022)
Archaeosomes are microorganisms that include archaebacterial membrane lipids		antioxidant distribution mechanism		(Alfadul and Elneshwy, 2010)
Nano cochleates are a type of nanomaterial that may be used to soy-based phospholipids		facilitate in the enhancement of processed food quality		(Abbas et al., 2009; Dib et al., 2022)

During food processing, storage and use, these functional components should be preserved from degradation. A delivery method for nutrients and supplements is an essential component that influences the effectiveness of food ingredients in the food industry (Abbas et al., 2009). The delivery method serves a variety of functions in transportation, including transporting functional materials to the intended action location. The functional components have also been shielded against chemical or biological deterioration in the delivery system to keep them active. Aside from that, the delivery method may be able to manage the delivery of the functioning substance, including the pace at which it is released and the precise environmental circumstances that cause it to be delivered. Additionally, the distribution strategy must be in line with the other components of the process as well as the physicochemical characteristics of the finished product, such as its appearance, texture, taste and shelf life. Because functional materials are so significant in delivery methods, so delivery methods have been implemented to encapsulate them, such as simple solutions, emulsions, colloids, biopolymer matrices and some others (Chau et al., 2007). Self-assembled nanotubes made from the hydrolyzed milk protein like α-lactalbumin may provide a novel naturally produced provider for nanoencapsulation of vitamins, nutrition and drugs (Graveland-Bikker and De Kruif, 2006). Encapsulation of food ingredients and additives is among the most prevalent applications of nanotechnology. Consumers may customize nano encapsulated foods to match their nutritional requirements and interests. Nano encapsulated food ingredients and additives/supplements provide protective barriers, taste and aroma concealment, sustained discharge and enhanced dispensability for water-insoluble food components and supplements/additives (Abbas et al., 2009). To provide nutrients, nano capsules can be introduced to foodstuff. Higher nutrient absorption may be achieved by adding nanoparticles to present foods. Another key use is the use of additives that are readily absorbed into the body and prolong the shelf life of commodities. Nanoparticle's colloids, emulsions and packed nano capsules do not settle, resulting in a longer product life and storage life. Dry processing (milling/grinding-materials are physically broken down into coarse particles using mechanical energy), high-pressure homogenization (decreasing fat globule size to enhance emulsion stability) or micofluidization (type of homogeneity wherein supplementary compartments is used to reduce the size and generate emulsions, improving texture and mouthfeel) and ultrasonic emulsification (employing high-intensity ultrasonic pulse capable of changing the characteristics of treated materials owing to cavitation's intense shear forces, pressure, and temperature) are the techniques used in the top-down method to prepare nanoparticles (Pathakoti et al., 2017; Singh, 2016). Its usage has benefited the making of salad dressings, yogurts, creams, chocolate, syrups and malted beverages, as well as fillings, flavor oil emulsions and icings (Kentish et al., 2008).

### 2.2 Food nanostructured materials in packaging and preservation

Food packaging is an important element of the food production process because it protects the food from external variables like temperature, humidity, microbiological infection, ambient gaseous mixtures, spill proofing and tempering. Packaging is a significant part of each stage of the food production process; yet the permeable/porous nature of traditional food packaging materials is a fundamental flaw. There are no packing materials that are completely impervious to water vapors and other ambient gases (Sharma et al., 2017). In terms of packaging, nanotechnology ensures food safety by preventing decomposition and loss of nutrients, resulting in a longer shelf life. Active packaging seems to have a significant role in food preservation, in addition to offering an inert shield to environmental circumstances. It mostly relates to packaging solutions that adapt to environmental changes. They work by releasing beneficial chemicals like antimicrobials or antioxidants, or by acting as gas scavengers. Antimicrobials, O_2_ scavengers and enzyme immobilization techniques are just a few of the packaging technologies that improve food stability due to such interactions. Another use of nanocomposites in active packaging is controlled-release packaging, in which they may be used as delivery methods to promote the absorption of functional supplements like nutrients, vitamins and probiotics in food (Graveland-Bikker and De Kruif, 2006). Some bionanocomposite constituents are aimed to enhance the functionality of typical food packaging, including mechanical strength, barrier performance and heat stability, by including antioxidants, strong antibacterial agents, enzymes and plant extracts (Youssef and El-Sayed, 2018). The advancement of eco-friendly and sustainable packaging has the potential to reduce the harmful ecological consequences produced by artificial packaging by means of biodegradable constituents, nanocomposite materials and plant extracts (Han et al., 2018). Because of the enhanced functionalities of nanostructured metals, nonmetals and their oxides, nanotechnology's applicability in food packaging is continually developing (Pereda et al., 2019). Direct uses of nanotechnology in foods and drinks are currently being investigated; however, indirect usage in food packaging have already been implemented (Smolander and Chaudhry, 2010). The numerous types of nano technologies and their applications were mentioned in Table 3 are used to innovate, improved, smart or intelligent and active food packaging, resulting in a shelf-life extension as a preservation.

**Table 3:** Application of several types of nano techniques for food packaging and preservation in food sector.

Nano-structured materials/particles	Techniques	Activity	Applications in Food Technology	Reference
Silver-Based	Nanoparticles: Categorize in inorganic and metal oxide nanomaterials	improved barrier and mechanical characteristics; yellowness, poor transparency, and heat stability; higher antioxidant activity; antibacterial activity that is effective against gram-positive and gram-negative bacteria	active packaging for food preservation in prolonging the food shelf-life and to control the pathogenic and spoilage microorganism/bacteria	(Arfat et al., 2017; Jafari et al., 2016; Ramachandraiah et al., 2017)
Zinc Oxide		powerful antibacterial agent; irradiation with UV-A had no influence on the mechanical characteristics of the nanomaterial produced; activated oxygen scavenging materials are used to prevent oxygen flow within packing containers	packaging highlights for food preservation emphasizes its antimicrobial impact and is utilized to extend the shelf life of fresh foodstuffs with inhibited foodstuffs from adhering together	(Esmailzadeh et al., 2016; Mizielińska et al., 2018)
Copper-Based		used to prevent bacteria, viruses, and fungus from growing; since of their large surface area, they were able to interface with cell membranes, and the antibacterial action was amplified; antimicrobial activity, permeation of water vapor, barrier characteristics, UV rays, and heat resistance	active packaging for food preservation in prolonging the food shelf-life and to control the pathogenic and spoilage microorganism/bacteria	(Almasi et al., 2018; Lomate et al., 2018; Shankar et al., 2017)
Titanium dioxide		offers several benefits, including being inexpensive, nontoxic, and photo-stable; gaining traction as a better photocatalyst particles for economical and power applications (water splitting, air or gas and water decontamination, antibacterial, and surfaces that clean themselves); antibacterial activity; polymer nanocomposites' mechanical characteristics have been enhanced; milk, cheese, and other various ​products are used as food whiteners	active packaging for food preservation in prolonging the food shelf-life and to control the pathogenic and spoilage microorganism/bacteria	(Roilo et al., 2018; Xing et al., 2012; Yadav et al., 2016)
Silicon dioxide		exhibits hygroscopic applicability by absorbing water molecules in food; moisture leakage is being decreased; serves as a food coloring, drying and anti-caking agents; typical particle size, large surface area, stability, biocompatibility, low toxicity, poor heat conductivity, and superlative insulation	active packaging for food preservation in prolonging the food shelf-life and to control the pathogenic and spoilage microorganism/bacteria	(Jones et al., 2008; Mallakpour and Nazari, 2018)
Nano-Clay and Silicate		increased overall volatiles, antioxidant activity, and organic acids; antibacterial activity	active packaging for food preservation in prolonging the food shelf-life and to control the pathogenic and spoilage microorganism/bacteria	(López-Rubio et al., 2019)
Polymer-Based: PVA (polyvinyl alcohol)	Nanoparticles: Categorize in organic biopolymer-based nanomaterials	improve the mechanical qualities associated with its suitable structure, as well as hydrophilic features such as solvent resistance, mechanical performance, biocompatibility, and high hydrophilicity; better antibacterial action, no cytotoxicity impact, and cell survival more than 90%	active packaging for food preservation in prolonging the food shelf-life and to control the pathogenic and spoilage microorganism/bacteria	(Gaaz et al., 2015; Sarwar et al., 2018)
Polymer-Based: PLA (polylactic acid)		demonstrates important features such as ​excellent mechanical capabilities, renewability, crystallinity, biodegradability, ​and processability	active packaging for food preservation in prolonging the food shelf-life and to control the pathogenic and spoilage microorganism/bacteria	(Sun et al., 2018; Swaroop and Shukla, 2018)
Polymer-Based: PHBV (3-hydroxybutyrate-co-3-hydroxyvalerate)		resistance to flammability, mechanical characteristics, ​heat stability, and ​rheological behavior have been enhanced; lead to improved water barrier and thermal characteristics	active packaging for food preservation in prolonging the food shelf-life and to control the pathogenic and spoilage microorganism/bacteria	(López-Rubio et al., 2019)
Polysaccharide-Based: Starch-Based		mechanical characteristics are strongly influenced, and this may minimize water vapor transmission and moisture absorption; integrated with multi-walled carbon nanotubes and enhanced by nanotube inclusion	active packaging for food preservation in prolonging the food shelf-life and to control the pathogenic and spoilage microorganism/bacteria	(Aqlil et al., 2017; Shahbazi et al., 2017)
Polysaccharide-Based: Cellulose-Based		nanocellulose's crystallinity index was lower than that of micro-crystalline cellulose; gram-negative and ​positive microorganisms were both suppressed by the anti-bacterial effectiveness	active packaging for food preservation in prolonging the food shelf-life and to control the pathogenic and spoilage microorganism/bacteria	(López-Rubio et al., 2019)
Polysaccharide-Based: Chitosan-Based		integrated with epicatechin gallate nano capsules and evaluated their antioxidant activities; integrated into packaging films; effective contact surface significantly reduced fruit microbiological deterioration	active packaging for food preservation in prolonging the food shelf-life and to control the pathogenic and spoilage microorganism/bacteria	(Buslovich et al., 2017; Liang et al., 2017)
Protein-Based: Zein-Based		strengthened mechanical and water moisture barrier characteristics while having no influence on film elongation; hydrophilicity and fractional free volume decreased; bacterial growth was considerably slowed; demonstrated an increase in tensile strength, a reduction in elasticity, and an initial rise in tensile strength	active packaging for food preservation in prolonging the food shelf-life and to control the pathogenic and spoilage microorganism/bacteria	(Gilbert et al., 2018; López-Rubio et al., 2019; Oymaci and Altinkaya, 2016)
Protein-Based: Whey Protein Isolate-Based		permeability of films to water vapor has been reduced; films' water resistance and barrier characteristics have been enhanced; reduced the degree of transparency	active packaging for food preservation in prolonging the food shelf-life and to control the pathogenic and spoilage microorganism/bacteria	(López-Rubio et al., 2019)
Nanocomposites with zinc oxide, pediocin, and silver coating	Nanocomposites	lipopolysaccharide degradation; damage the bacterial DNA in an irreversible way; assist in the fight against microorganisms	improved food packaging composition with distinctive characteristics (antimicrobial agent)	(Sundaramoorthy & Nagarajan, 2022)
Polymer & nanoparticles (nano clay)		gas barriers are used to reduce carbon dioxide leaks from carbonated beverage bottles	improved food packaging composition with distinctive characteristics (antimicrobial agent)	(Yotova et al., 2013)
Nanolaminates (nanoencapsulation)		meats, cheeses, ​vegetables, fruits, ​and baked products are all coated in it	improved food packaging composition with distinctive characteristics (antimicrobial agent)	(Miranda et al., 2022)
Garlic oil nanocomposites coated with PEG		eliminate insects that commonly infects packaged food items at shops	improved food packaging composition with distinctive characteristics (antimicrobial agent)	(Miranda et al., 2022)
DS13 Top Screen & Guard IN Fresh		scavenge ethylene molecules to support in the ripening of fruits and ​vegetables	improved food packaging composition with distinctive characteristics (antimicrobial agent)	(Ghosh et al., 2022)
Nanocor		to restrict carbon dioxide from leaking from a drink, it is used in the production of plastic beer bottles	improved food packaging composition with distinctive characteristics (antimicrobial agent)	(Ashfaq et al., 2022)
Aegis		assist in the retention of carbon dioxide in carbonated beverages by acting as oxygen scavengers	improved food packaging composition with distinctive characteristics (antimicrobial agent)	(Yotova et al., 2013)
Immobilization of enzymes		greater surface area and quicker transmission rates are enabled	improved food packaging composition with distinctive characteristics (antimicrobial agent)	(Kumar and Kirupavathy, 2022)
PAC Nano Ceram		assists in the fast absorption of undesirable elements that can generate a bad smell and an unpleasant taste	improved food packaging composition with distinctive characteristics (antimicrobial agent)	(Sarkar et al., 2022)
Bio nanocomposites (cellulose & starch)		deposition substances for packaging purposes have been shown to be efficient	improved food packaging composition with distinctive characteristics (antimicrobial agent)	(Pradhan et al., 2015)
Imperm (nylon)		oxygen scavenging is the purpose of this mechanism	improved food packaging composition with distinctive characteristics (antimicrobial agent)	(Thirumurugan et al., 2013)
Durethan (polyamide)		provides rigidity to fruit juice paper packaging jars	improved food packaging composition with distinctive characteristics (antimicrobial agent)	(Davis et al., 2013)
Nano biosensors	Nano sensors	bacteria and viruses ​are being identified	smart (intelligent) food packaging in prolonging the shelf-life and to control and identify the pathogenic and spoilage bacteria	(Coles and Frewer, 2013)
Nano-smart dust		investigation of all forms of pollutants in the environment	smart (intelligent) food packaging in prolonging the shelf-life and to control and identify the pathogenic and spoilage bacteria	(Coles and Frewer, 2013)
Abuse indicators		evaluation of whether the target temperature was obtained	smart (intelligent) food packaging in prolonging the shelf-life and to control and identify the pathogenic and spoilage bacteria	(Su et al., 2013)
Nano barcodes		evaluation of the agricultural product's quality	smart (intelligent) food packaging in prolonging the shelf-life and to control and identify the pathogenic and spoilage bacteria	(Coles and Frewer, 2013)
Interferometry with reflections		infections of packaged foodstuffs ​with *E. coli* were detected	smart (intelligent) food packaging in prolonging the shelf-life and to control and identify the pathogenic and spoilage bacteria	(Bashir et al., 2022)
Indicator of the entire temperature history		identification of temperature variations in frozen foodstuffs; temperature changes over time are observed	smart (intelligent) food packaging in prolonging the shelf-life and to control and identify the pathogenic and spoilage bacteria	(Hu and Miao, 2022)
Indicator for partial temperature history		when the temperature rises over a particular threshold, the time-temperature history is amalgamated	smart (intelligent) food packaging in prolonging the shelf-life and to control and identify the pathogenic and spoilage bacteria	(Su et al., 2013)
Plasmon-coupled emission biosensors on the surface (with Au)		pathogenic microorganism identification	smart (intelligent) food packaging in prolonging the shelf-life and to control and identify the pathogenic and spoilage bacteria	(Senturk and Otles, 2016)
Biosensor arrays, nano-test strips, electronic noses, and nanocantilevers are among the technologies being developed		when it comes into touch with any indication of deterioration in the foodstuff, it changes color	smart (intelligent) food packaging in prolonging the shelf-life and to control and identify the pathogenic and spoilage bacteria	(Biswal et al., 2012)
Smart biosensors and biomimetic sensors (biomimetic membranes and proteins)		assist in the identification and eradication of infections by acting as fictitious cell surfaces; mycotoxins and a variety of other hazardous chemicals are detected	smart (intelligent) food packaging in prolonging the shelf-life and to control and identify the pathogenic and spoilage bacteria	(Coles and Frewer, 2013)
DNA and single-walled carbon nanotubes		pesticide residues on the exterior of ​vegetables and fruits ​are detected; crop's development requires constant monitoring of the soil's condition	smart (intelligent) food packaging in prolonging the shelf-life and to control and identify the pathogenic and spoilage bacteria	(Sozer and Kokini, 2009)
Nano sensors made of metals (platinum, palladium, ​and gold)		light, humidity, heat, gas, and chemical changes are observed and converted into electrical impulses; observation of any abnormalities in the food's color; toxins like aflatoxin B1 have been identified in milk; identification of any gases generated because of deterioration	smart (intelligent) food packaging in prolonging the shelf-life and to control and identify the pathogenic and spoilage bacteria	(Kang et al., 2007; Meetoo, 2011)
Time-temperature indicator/integrator iSTrip		thermal record is used to detect food deterioration	smart (intelligent) food packaging in prolonging the shelf-life and to control and identify the pathogenic and spoilage bacteria	(Li et al., 2005)
Immunosensors made of cerium oxide and nanocomposites made of chitosan		numerous toxins, including ochratoxin A, were revealed	smart (intelligent) food packaging in prolonging the shelf-life and to control and identify the pathogenic and spoilage bacteria	(Mousavi and Rezaei, 2011)
Polyaniline with carbon black		microorganisms that infest food are identified; diagnosis of infections that are transmitted by food; carcinogens in food items are being revealed	smart (intelligent) food packaging in prolonging the shelf-life and to control and identify the pathogenic and spoilage bacteria	(Biswal et al., 2012; Vidhyalakshmi et al., 2009)
Silicon nanowire transistors with carbon nanotubes		cholera toxin and staphylococcal enterotoxin B identification	smart (intelligent) food packaging in prolonging the shelf-life and to control and identify the pathogenic and spoilage bacteria	(Mousavi and Rezaei, 2011)

Moreover, researchers from a variety of disciplines have been drawn to nanotechnology's great potential for developing innovative and attractive materials for food packaging technologies. The two primary functionalities of nanocomposite materials for food packaging that have been described are improved and smart (intelligent and active) food packaging (Sharma et al., 2017): (1) improved food packaging-the addition of nanoparticles to bio nanostructured materials enhances strength and durability properties such as flexibility, gas impediment abilities (resistance to the flow of CO_2_, O_2_ and aroma compounds) and durability under a variety of temperature and humidity situations (Youssef and El-Sayed, 2018); (2) Smart packaging not only monitors (intelligent) but also interacts with and preserves the food it contains (active). As a result, these packages are both at the same time active and intelligent. Intelligent packaging does not have to preserve the food, instead, it just monitors specific characteristics of the food packaging. 2(a) intelligent food packaging-in associated with detailed responses and promotion on the proven quality of packaged foodstuffs, it serves as a protection against deception and counterfeit goods, as well as an indication of the condition of visibility to certain unfavorable circumstances like inadequate temperature levels or excessive O_2_ saturation (Suh et al., 2016; Wang et al., 2017); 2(b) active food packaging-protects and preserves food by processes triggered by innate and attained factors (antioxidant and antimicrobial activity of packaging materials itself) and reduces food waste by prolonging shelf life (Wyrwa and Barska, 2017). The utilization of nanoparticles in food manufacturing introduces up a plethora of potentials, which include either improving the authentic polymer capabilities (barrier or mechanical characteristics) or introducing novel features (active or biologically active packaging, sensing and controlling) based on nano additives intrinsic characteristics.

**Figure 2:**
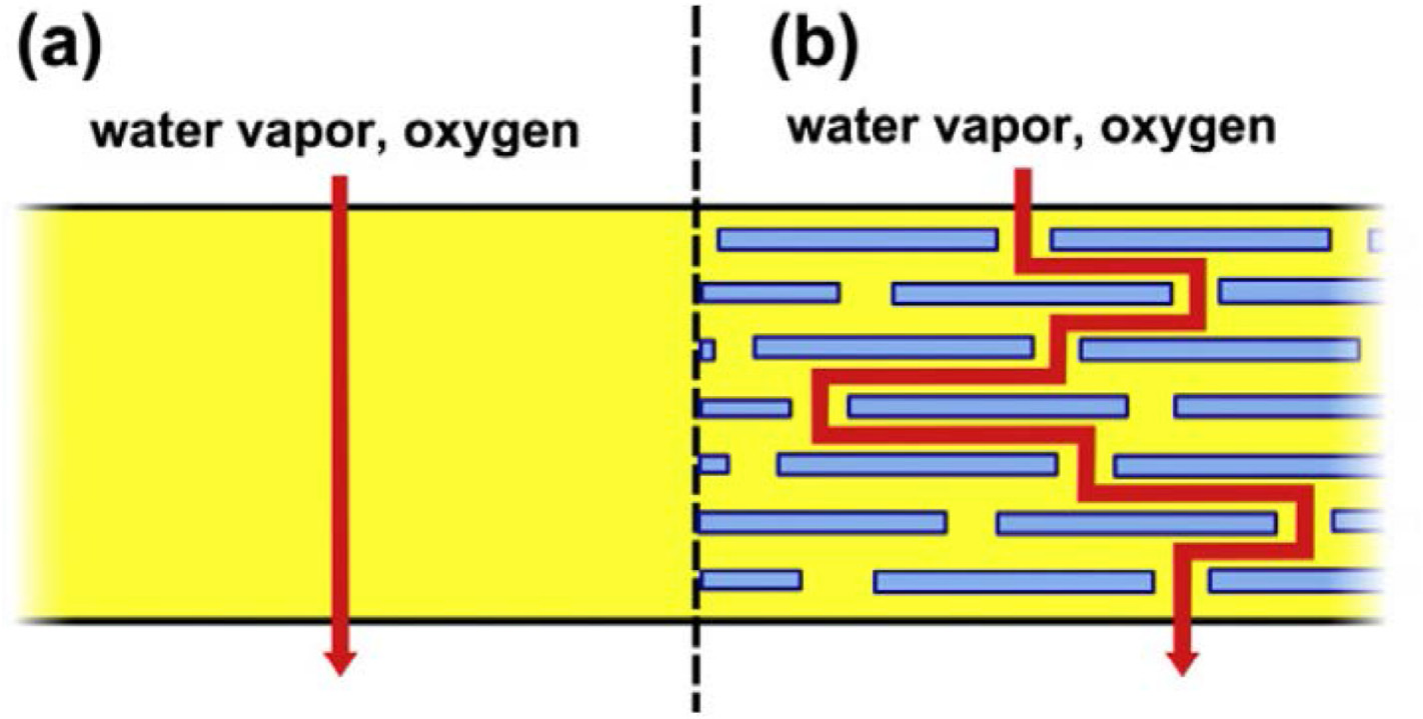
In the case of a polymer-only film (a), in a nanocomposite (b). Reprinted with the permission of Ref (Duncan, 2011).

In improved food packaging implications, thermal resistance and barrier characteristics of nanocomposite films using graphene nanoplates have been described (Ramanathan et al., 2008). Carbon nanotubes and nanofibers are used in food packaging because of their electrical and mechanical properties, although their usage is restricted owing to their expensiveness and difficulty processing diffractions (Arora and Padua, 2010). To enhance barrier and mechanical characteristics, nano clays containing montmorillonite nanoparticles in various starch-based composites (biodegradable polymers) were synthesized (Graveland-Bikker and De Kruif, 2006). When integrated into a polymer, the most widely used nano clay substance montmorillonite (bentonite) is utilized to create gas impediment characteristics and can inhibit gas penetration. It is also readily accessible and reasonably priced. Biodegradable nanocomposites made from starch clay have been used in a variety of applications, involving food packaging (Chen and Evans, 2005; Cyras et al., 2008; Huang et al., 2004, 2005; Park et al., 2002). Finally, due to the lower amounts of polymer that must be utilized to provide packaging materials with equal or even excellent mechanical characteristics, polymer-nanocomposites should give the food packaging sector improved downgauging opportunities, as well as cost savings and waste reductions. When a nanofiller is distributed inside the bio-compatible polymer PLA (polylactic acid), the PLA bio nanocomposite has a quicker rate of biodegradation than PLA that does not contain such additives (Sinha Ray et al., 2002). Nano-sized fillers dispersed in a polymer matrix have two distinct effects on the barrier characteristics of a homogeneous polymer film where the first method involves constructing a tortuous pathway for gas (O_2_, CO_2_ and vapor) diffusion (Choudalakis and Gotsis, 2009). Since the admixtures are basically impervious inorganic crystals, air molecules must disperse around them instead of following a straight-line channel perpendicular to the film surface (mean) (Duncan, 2011). In the presence of fillers, therefore, the average pathway for the exchange of gases through the film is lengthier shown in Figure 2 where the tortuous pathway enables the manufacturer to achieve higher efficient film densities while using less polymer. The "tortuous pathway" formed by exfoliated clay nanocomposite embedded into a polymer matrix film. In the case of a polymer-only film (a) the typical migration path for diffusing gas molecules is perpendicular to the film alignment; In a nanocomposite (b) diffusing molecules must traverse through impermeable particles and across contact zones with permeability properties that are different from those of the virgin polymer. The tortuous pathway lengthens the mean gas diffusion length, extending the shelf life of perishable foods (Duncan, 2011). The impact of scattered nanoparticles on gas diffusion average pathway length has been analytically predicted and the most basic model, introduced by Nielsen, implies the granules are distributed uniformly across the structure, resulting in the formation of rectangular patches of regular shape and that the tortuosity of the pathway is the only variable affecting gas flow coefficient (Duncan, 2011). The gas permeability is calculated by using Nielsen model Eq. (1):

(1) $$\frac{K_{composite}}{K_{matrix}}=\frac{1-\varphi }{1+\frac{\alpha }{2}\varphi }$$

where K values indicate the permeabilities of the composite material and matrix in the absence of filler, α is aspect ratio of the individual filler particles ($\frac{length}{width}$) and φ is the volume fraction of filler. The barrier efficacy is anticipated to rise as the particles grow more anisotropic or plate-like in form, according to this Eq. (1), which has been empirically validated (Choudalakis and Gotsis, 2009). In general, the Nielsen model is only applicable for low loading percentages (φ<10%), since greater loadings cause particle aggregation, which decreases the average particle aspect ratio (Choudalakis and Gotsis, 2009).

In smart (intelligent) food packaging, nanodevices or nano sensors are used in combination with polymers to detect food pathogens and contaminants/chemicals/toxins throughout storage and transportation operations (Lerner et al., 2011). Moreover, smart food packaging protects the food package's integrity and the product's validity. These gadgets can also keep tracking of duration, temperature and date of expiry. According to several recent studies, nano sensors can identify food spoilage organisms like pathogens, contaminants and toxins in food packaging (Li and Sheng, 2014). Nano sensors have also been designed for food analysis, assessments, evaluations, drinking water, aromas and medical diagnostics, and nanoparticles can be used as nanostructured transducers in biosensor systems (Pathakoti et al., 2017).

**Figure 3:**
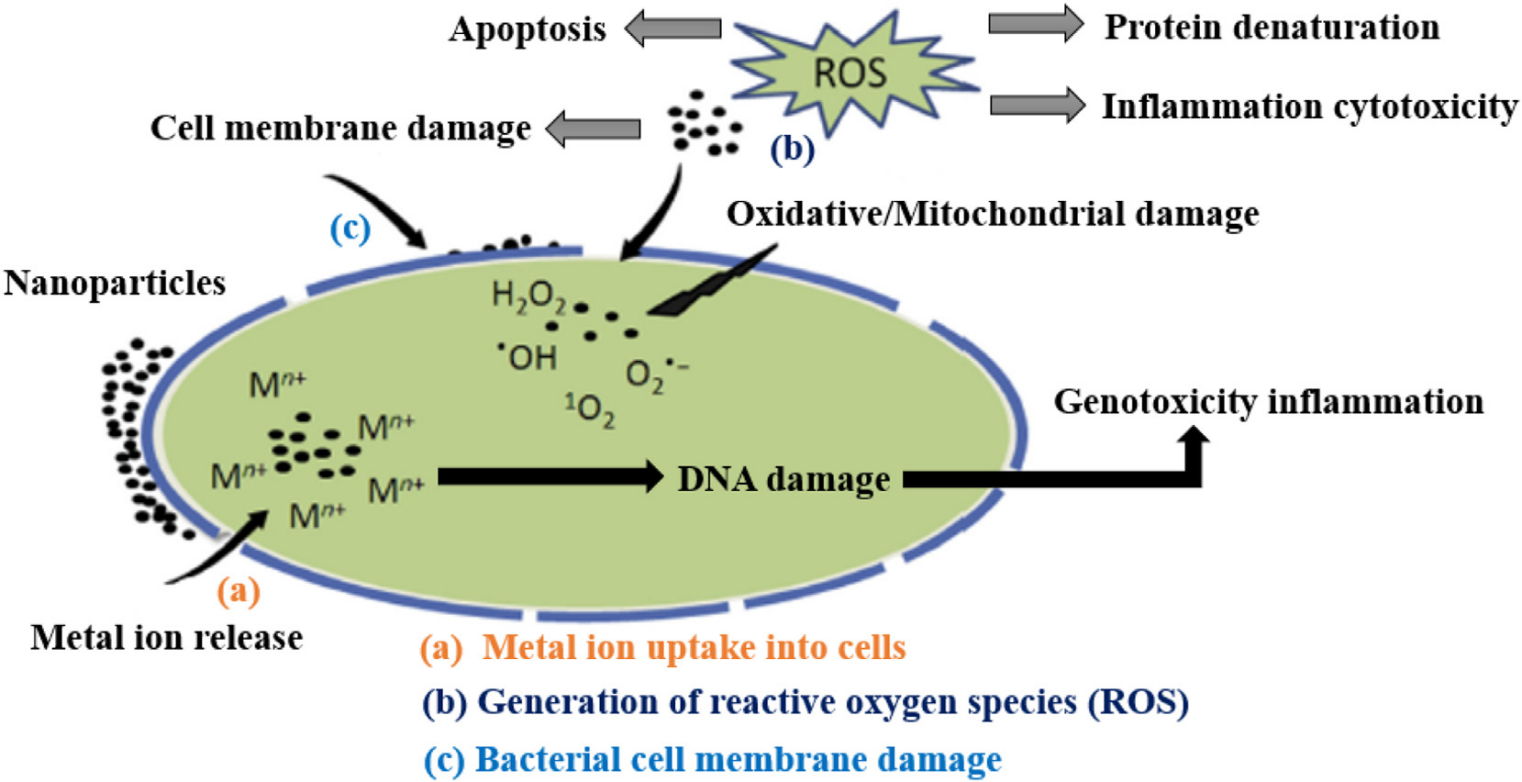
Schematic diagram of nanoparticle toxicity and antibacterial mechanisms. ROS = reactive oxygen species. Adapted from (López-Rubio et al., 2019; Naidu et al., 2015; Pathakoti et al., 2017).

As a smart (active) food packaging ZnO, Cu, Cu_2_O, CuO, TiO_2_ and Ag-based nanofillers are utilized for food packaging using antibacterial nanocomposites, while TiO_2_ and SiO_2_ based nanofillers are employed to be used in self-cleaning surfaces (Lee, 2010). The most common nanoparticles are silver nanoparticles, which are efficient against a range of microorganisms (Becaro et al., 2015). The mechanisms of silver antibacterial agents involve cellular surface adhesion, breakdown of cellular membranes, DNA damage and the release of silver ions (Dallas et al., 2011; Lee, 2010). Metal nanostructures' antimicrobial activity is largely determined by variables including shape, size, surface area, penetration of particles and chemical bioactivity (Diaz-Visurraga et al., 2010). Some investigations have shown that nanostructured materials may permeate both the exterior and inner cell membranes of microorganisms (Dasari et al., 2013; Díaz-Visurraga et al., 2011; Pathakoti et al., 2013). Metal ions absorption causes depletion of intracellular ATP (Lok et al., 2006), production of ROS causes cells harmed by oxidative stress (Fu et al., 2014) and bacterial membrane damage (Amro et al., 2000) are three primary mechanisms of microorganisms toxicity and cytotoxicity of metal nanomaterials illustrated in Figure 3. Superoxide anion is a kind of free radical (O_2_∗^−^) and hydroxyl radical (∗OH), in addition to non-radical compounds like hydrogen peroxide (H_2_O_2_) and singlet oxygen (^1^O_2_), are examples of reactive oxygen species (ROS) (Fu et al., 2014). Titanium dioxide is utilized as a photocatalyst, pigment, UV blocker and antibacterial agent that is efficient against microorganisms that cause food deterioration, as well as in food packaging (Chawengkijwanich and Hayata, 2008; Lee, 2010; Yemmireddy and Hung, 2015). As a result of lipid peroxidation and oxidative stress caused by the production of ROS under visible and UV light (photocatalytic activity), cells die (Matějka and Tokarský, 2014; Pathakoti et al., 2014).

The use of nanotechnology has improved the taste, absorption, and bioavailability of nutraceuticals and additives/supplements that contain nanosized components and nano additives such as antimicrobials, nutrients, antioxidants, and preservatives (Momin et al., 2013). Because of the changed respiration mechanism, vegetables and fruits coated with nano silver stay active during transit and storage. For improved absorption and bioavailability, nanoparticles of heavy metal are being investigated like a component of consumable protective coatings (edible or biodegradable coating materials). Lycopene, phytosterols and β-carotene are among the nutraceuticals included in the carriers to prevent cholesterol buildup (Mozafari et al., 2006). Nanoencapsulation is the technique of packaging materials at the nanoscale using nano capsules to offer finished product functionality, such as controlled core release. Encapsulated substances have numerous advantages, including a longer shelf life, improved stability, continuous delivery of several active components and pH-triggered controlled release. A nano delivery system incorporates functional components including antioxidants, preservatives, vitamins, carotenoids, probiotics, omega-3 fatty acids, lipids, peptides, proteins and carbohydrates (Elliott and Ong, 2002). Because these foods are not utilized in their natural state, their functioning and stability are improved. Lipid-based nanoencapsulation has the effectiveness to increase food solubility, stability and bioavailability while also avoiding undesired association with other food materials. Among the most effective lipid-based antioxidant transporters are nanoliposomes and nano cochleates. Nanoliposomes also contribute in the supply of nutrients, nutraceuticals, enzymes, antimicrobials, vitamins and additives in a regulated and precise manner (Reza Mozafari et al., 2008). Food preservation treatments based on nanotechnology have greatly increased detection capability and decreased the burden of traditional screening. It can also cover up undesirable aromas produced by numerous food processing unit activities (Davies, 2007).

## 3 Health risk associated with toxicity and safety assessment with certain aspects of nanoparticles in food

Numerous commodities developed because of the ever-evolving area of nanotechnology represent a severe threat to the public's health. Although many food components and constituents contain nanostructure in origin, the introduction of synthesized nanoparticles into the food system may result in the deposition of harmful pollutants in foodstuffs, posing a health risk to humans as well as consequences to affect the environment. Food packaging nanoparticles have been established to improve the barrier and mechanical characteristics of conventional and bio-based packaging materials, as well as to offer new active and smart activities in which packaging materials have active or smart components that are designed to discharge or absorption chemicals into, onto, or out of the packed foodstuff or external area, or to give the required details about their usage circumstances (Dudefoi et al., 2018). As a result of inadequate packing performance, consumption of foods in touch with nano packaging may provide an exposure pathway and represent a substantial health threat, particularly in terms of toxicity and ecotoxicity because of particle nanomaterials being transmitted from the packaging into foodstuff. Increased production of ROS by nanostructured materials is predicted to induce cell damage or oxidative stress, in contrary to their positive uses as antimicrobials and antioxidants. Nanotoxicity can therefore cause DNA damage, uncontrolled cell stimulation, cell mobility changes, toxicity, cytotoxicity, apoptosis and cancer formation (Fu et al., 2014). The toxicity of the nanoparticle employed, the type of the packing matrix, the extent of migration and the absorption rate of the specific foodstuff would all play a role in this impact (Cushen et al., 2012). The evidence of health risks associated with absorbing nanoparticles is growing day by day and nano-based foodstuffs have a negative impact on health due to excessive intake, bioaccumulation and overactivity, as well as the dangers and hazards that come with them (Cushen et al., 2012; Jovanović, 2015; Rasmussen et al., 2010; Yang et al., 2010). The possible risks of nanostructured materials may be assessed by the locations of invasion, subsequent deposition and transportation of nanomaterials in the body (Chau et al., 2007). Associated with exposure to greater quantities of these kind of compounds by respiratory or skin absorption may pose severe security issues, necessitating more study and risk assessment, particularly in terms of long-term cytotoxicity and toxicity (He and Hwang, 2016). According to certain research, packaging materials containing nanomaterials can migrate into food and be consumed by consumers, yet there is little information on their toxicity (Echegoyen and Nerín, 2013). However, these nanoparticles may gather in various organs in humans, including the stomach, kidneys, liver, small intestine, lungs, spleen and major distribution organs; additionally, difficulties like lung damage, kidney diseases and hepatic injury may happen as a consequence of a single oral dose of ZnO nanoparticles, and the gastrointestinal tract offers a chance for nanoparticle ingestion which may readily pass through biological barriers and enter the circulatory system (Esmaeillou et al., 2013; McClements and Xiao, 2017). Table 4 highlights the consequences of nanotechnology in the biological system in the application of food systems. The use of titanium oxide and its discharge can have an impact on people and nature, raising the risk of harm to the environment and human health (Yang et al., 2014).

**Table 4:** Health impacts and risks of using nanotechnology in food systems.

Application Systems	Harmful Materials	Nano emulsion	Risk Factors	Reference
Food processing	Carbohydrates, proteins, and lipids are all surfactants	Organic nanostructured materials ​that are digestible	breakdown ​of proteins and cardiovascular disorders	(Mills and Hazafy, 2009; Pilli et al., 2022)
	Ag nanomaterials	Inorganic nanostructured materials ​that are indigestible	chromosomal distortion is a type of chromosomal abnormality; mitochondrial dysfunction and DNA damage	(Dhara and Nayak, 2022)
Food packaging	Carbohydrates, proteins, and lipids are all surfactants	Organic nanostructured materials ​that are digestible	disruption to cellular and bioaccumulation	(Ibisanmi, 2022)
	Nanotubes of carbon	Organic nanostructured materials ​that are digestible	toxic to the skin and lungs	(Yayehrad et al., 2022)
	Ag nanomaterials	Inorganic nanostructured materials ​that are indigestible	increasing the amount of ROS produced; decreasing the amount of ATP in the body	(Francesconi et al., 2022)
Food preservation	Carbohydrates, proteins, and lipids are all surfactants	Organic nanostructured materials ​that are digestible	overweight/obesity	(Corb Aron et al., 2021)
	Ag nanomaterials	Inorganic nanostructured materials ​that are indigestible	carcinogenic, genotoxic, and cytotoxic are all terms used to describe substances that possibility of causing cancer	(Ezhilarasi et al., 2013)

There are apparently just a few studies on the health impacts of nanoparticles in foodstuff. Risk associated with a nanomaterial is determined by its chemical make-up, physical and chemical properties, interactions with tissues, and exposure levels. There are a few broad difficulties (problem formulation) that need to be addressed early on before assessing a nanomaterial that is suggested for usage in the food/feed chain. To recognize a substance as a nanomaterial, physicochemical characterization is required. The strength of a nanomaterial's nanoscale characteristics is inextricably connected to its unique qualities or effects. When a nanomaterial loses these properties, such as via deterioration or disintegration, it is anticipated to behave similarly to its non-nanomaterial counterpart if one exists. Because of this, safety concerns with orally ingested nanomaterials are mostly focused on those that may escape the digestive system, potentially allowing nanoparticles to be translocated to organs and other tissues or producing local detrimental effects in the gastrointestinal tract (*in vitro* digestion models have been extensively examined), food contamination, and other potential risks (Dall’Asta et al., 2010), food components (Blanquet-Diot et al., 2009; Tydeman et al., 2010). The circumstances of the gastrointestinal tract are simulated in these models (including mouth, stomach, and gut). The distinctions between these models relate to the degree to which physiology is represented, ranging from extremely simple to comprehensive models that use static or dynamic settings and include or exclude enzymes, bile salts and other factors. Furthermore, the physiology simulated may differ between models: starved vs. fed situations, newborn vs. adult. Although there is still hardly a piece of information on the carcinogenicity, genotoxicity, or teratogenicity of nanoparticles in food, the United Nations Food and Agriculture Organization (FAO), the World Health Organization (WHO) and the European Union (EU) have all stated that the safety of nanoparticles in food must be reinforced (Magnuson et al., 2013). The European Food Safety Authority (EFSA) has revised its Guidance on risk assessment of nanoscience and nanotechnology applications in the food and feed chain, as well as human and animal health. It includes new foods, food/feed additives, food contact materials and pesticides, among other application areas under EFSA's purview. The amended guideline, now known as Scientific Committee Guidance on nano risk assessment (SC Guidance on Nano-RA), has explored significant scientific investigations that give insights into nanomaterials' physicochemical characteristics, exposure assessment and hazard characterization, as well as their applicable regions (Committee et al., 2021). There are several concerns about nanomaterials, nanoparticle fractions and materials having nanoscale features that might provide a risk to consumers. A Scientific Consultation has been issued by the Scientific Committee on Consumer Safety (SCCS) (SCCS, 2019), emphasizing the characteristics of nanoparticles that might cause concern in terms of safety, wherein they could be referred to risk assessment. Recognizing the paucity of any strict guidelines for determining the overall level of safety concern for certain nanopasrticles, the Consultation specified the number of characteristics, each of which should raise a level of caution about the nanomaterial's safety. A grading approach was also established in the Consultation, in accordance with expert opinion to ascribe an identifiable grade for nanomaterials risk potential (Brand et al., 2020). A conclusion was stated that degradation, genotoxicity, accumulation and immunotoxicity are all components of toxicokinetics and human hazard assessment that are most likely impacted by nano-specific features of the substance. As a result, these considerations should take precedence in the hazard assessment of nanomaterials (Dekkers et al., 2016). First, we should focus on the safety assessment of food or foodstuff prior to the implementation of nanotechnology in the food system to reduce the toxicity with health risks associated with nanoparticles which are following some criteria:

• Standardize identification and measurement of nanoparticles in food commodities or foodstuff.
• Standardize administration guidelines of nanoparticles in food commodities or foodstuff.
• Determine the consumer's maximum exposure to nanoparticles in food commodities or foodstuff.
• Design the dietary pharmacokinetic model for nanoparticles in food commodities or foodstuff.
• Implement a standardized technique for analyzing nanoparticles in food commodities or foodstuff.

**Figure 4:**
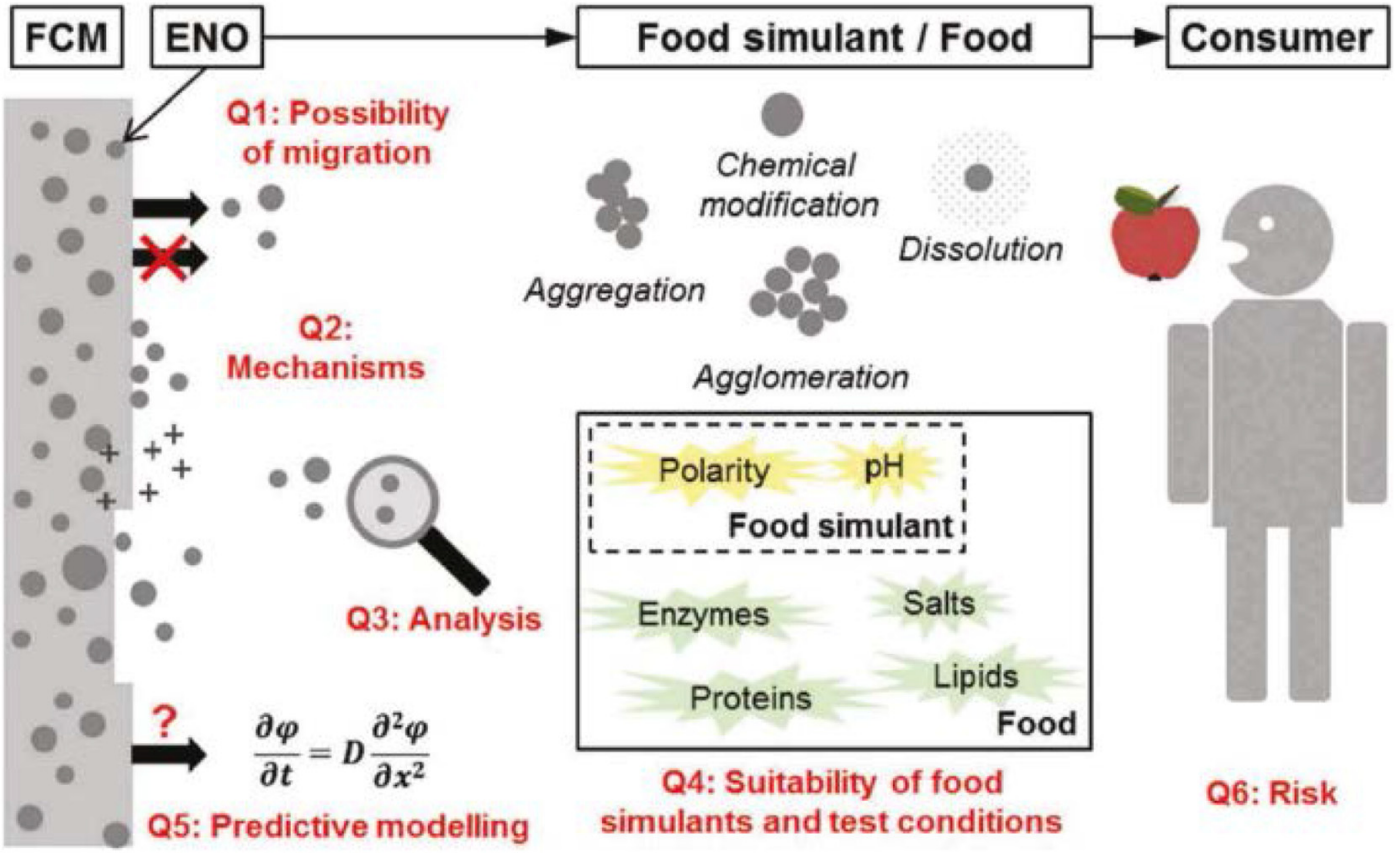
Illustration of the six outstanding problems concerning nanoparticle dispersion from polymer-based foodstuff packaging films addressed (FCM = food-contact materials, ENO = engineered nano-objects). Reprinted with the permission of Ref (Jokar et al., 2017).

Nanoparticles can migrate to packaged food in conceptually, however, migration tests and risk assessments are currently lacking and migrations into foodstuff may be regarded as a mass transfer process whereby lower molecular mass components are trapped in packaging and subsequently released into the packaged substrate, as a result, it was thought to be a diffusion process that could be characterized using Fick's second law, as a consequence, one of the most significant phases in the creation of innovative food packaging materials is migration studies, which explores the possibility of any unwanted or hazardous substances transferring to foodstuffs in general and in particular terms (Souza and Fernando, 2016). Figure 4 shows the six outstanding problems that arise during nanoparticle (ENO) dispersion from polymer-based foodstuff packaging materials to consumers (Jokar et al., 2017). Specifically, it was discovered throughout the research that numerous experimental investigations had failed to provide a convincing response on the probability of nanoparticles migration from food packaging materials to foodstuffs, and they theorized that this was due to the absence of adequate analytical techniques for identifying nanoparticles with lower content and smaller diameters that strongly recommended that investigations that showed no evidence of migration give information on the detection limit of the assessment, which should include particle mass or number concentration as well as particle size (Jokar et al., 2017; López-Rubio et al., 2019). Engineered nano-objects (ENO) have gained widespread use in the food industry due to their antibacterial, antimicrobial, water resistance, and protective qualities. Unfortunately, despite the widespread use and distribution of ENO, their toxicity and potential for hazard have not been properly explored. However, ENO can be customized to meet specific needs and chosen for their minimal toxicity to individuals. They may also be used to identify contaminants on the spot. All these characteristics make ENO viable environmental pollution remedies (Ramakrishnan et al., 2021). Nevertheless, some safety requirements in terms of permitted ranges or limitations of nanoparticles used in food applications must be stated, and environmental rules may also raise concerns about the use of nanomaterials in food packaging since they will be disposed of in some manner. A collection of information on rules governing nanotechnology-based food applications has been gathered (Gergely et al., 2010; Hodge et al., 2014). As a result, further research is required to determine the potential toxicity of nanostructured materials used in the food industry, as well as food scientists, researchers and consumers all need a global knowledge-sharing framework where they can consult and resolve all aspects of implementation, consumption, disposal and long-term consequences. It will enable to improve in research and applications in the food area.

## 4 Future perspectives and challenges of nanotechnology in food

Whereas scientific advancement in the implementation of nanotechnology to food sectors has been tremendous, progress in nanotechnology linked with nanostructures has been much less. Although nanotechnology offers a lot of promises in the food industry for manufacturing novel commodities and procedures, there are a lot of barriers to solve. As food nanotechnology studies advance, societal worries regarding the safety of nanotechnology commodities for human consumption and use grow (Rastogi, 2012). Consequently, before nano food products are available commercially, a thorough study of possible hazards to human health is required. Nevertheless, there has yet to be created a general guideline for the assessment of the safety of nanomaterials in foodstuffs. The main issue is to develop edible delivery systems that are cost-effective to produce and safe for human ingestion (Dupas and Lahmani, 2007). To guarantee the healthiness of foods, migration and absorption of nanoparticles from packaging materials into foodstuffs is a major problem. At the nanoscale, materials act quite differently, yet we still have a vague awareness of how to investigate those. Nanoscale functions and toxicities of nanomaterials will be better understood, which will improve their pragmatic usage safety standards.

Furthermore, it was hard to determine nanoparticles migrations using prediction models that solely included diffusion-based migrations. In general, three sub-processes may be differentiated in nanoparticles migration: (i) due to a concentration difference, the molecule diffuses into the polymer to reach foodstuffs; (ii) at the food-packaging contact, the molecule is dissociated from the polymer and then absorbed by the foodstuff; (iii) due to a concentration differential, the molecule diffuses through the foodstuff (Jokar et al., 2017). Nevertheless, there was a distinct paucity of information on the probable release mechanisms of the nanoparticles that had been observed. Because there were many influences of physio-chemical characteristics of nanoparticles on gastrointestinal absorption, including morphology, composition, surface properties, charge and aggregation state, as well as functional ingredients, the question of consumer risk associated with migrating nanoparticles from food packaging was likely more complicated than other questions (McClements et al., 2017), as a result, these are obviously areas that need to be investigated more in the future. Nanoparticle consequences, possible danger and related toxicological risks, as well as environmental issues, must all be considered. Nanoparticles have been observed to overcome the biological border and penetrate cells and organs (Cha and Chinnan, 2004). Nanoparticles synthesis by various chemical techniques has negative consequences and produces harmful non-eco-friendly by-products that pollute the environment (Singhal et al., 2011). As a consequence, in addition to public demand and popularity, a comprehensive risk assessment strategy, biosafety, regulatory regimes and public concerns must all be taken into account during the food processing, production, packaging and public consumption of nano-based foodstuffs (Bajpai et al., 2018; Shi et al., 2013; Yu et al., 2012). Furthermore, before to implementation and manufacturing of antimicrobial nanoparticles with sustainable alternatives, in vitro and in vivo research including nanoparticle interactions with biological organisms are required (Das et al., 2011). The Food and Drug Administration concluded that any distinctive features and characteristics transmitted by nanotechnology should be included in studies of safety, efficacy, public health impact, or regulatory status of nanotechnology commodities (Guidance, 2011). The European Commission has indeed compiled legislated entirety in this path with technology and the advancement referencing nanoparticles, as well as smart (intelligent) and active materials to assert that technological innovations, which are based on nanostructured materials size with physical and chemical characteristics that vary considerably from those at a much greater scale, should be identified (Baiguini et al., 2011). On this framework and due to a lack of understanding regarding their potential toxicity (oral exposure) nanomaterials have gotten fewer considerations than the cutaneous or inhalation routes (Jo et al., 2016). When nanotechnology-specific legislations are established to address the numerous safety concerns that this technology raises, it has the potential to govern the whole food processing sector.

**Figure 5:**
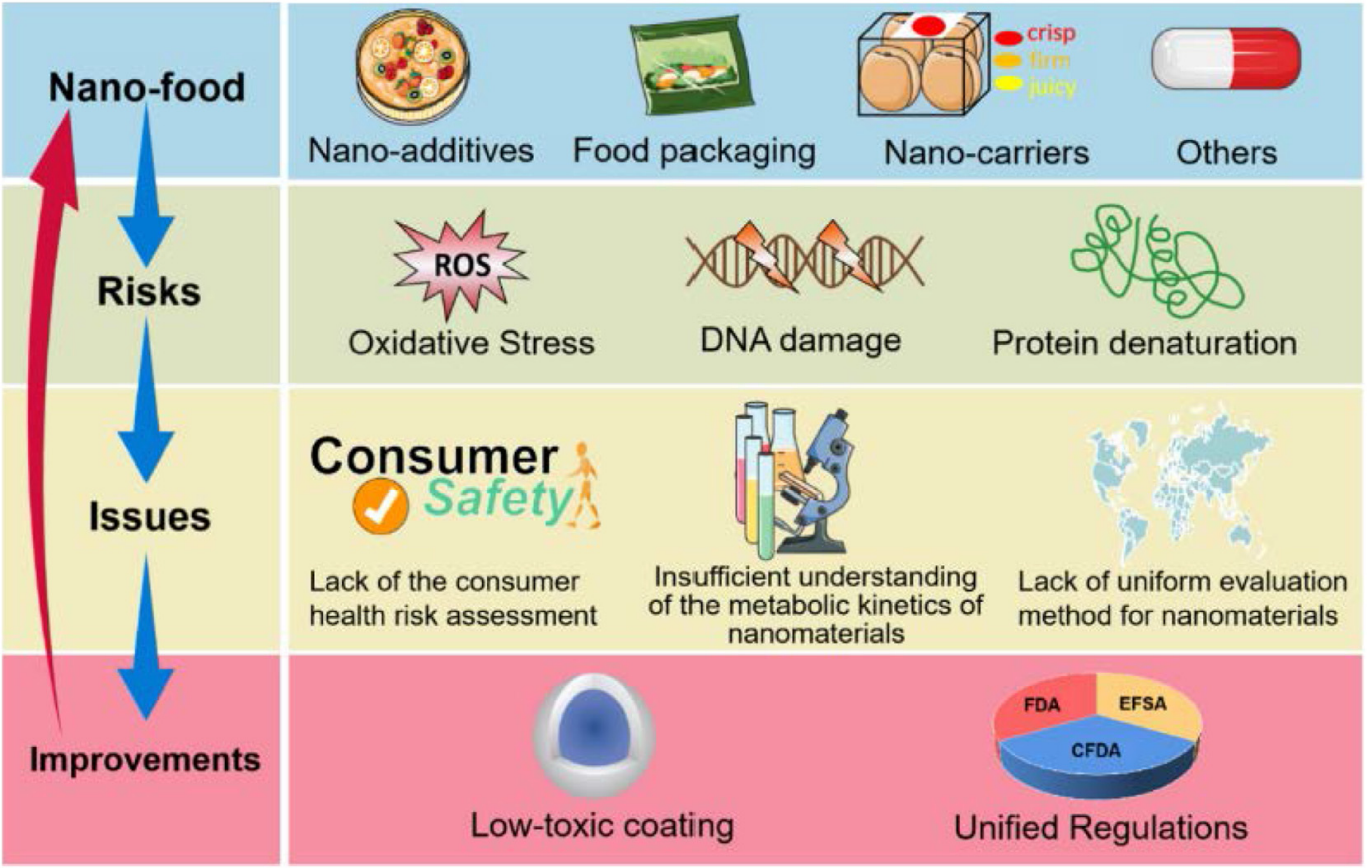
Illustration of issues or challenges with nanoparticles in foodstuff and how to solve. Reprinted with the permission of Ref Wang et al. (2021).

Now it is the high time to work on improving nanoparticles in foodstuffs safety evaluations based on exposure and toxic response mechanisms which mechanism followed in Figure 5. Biological consequences of nanoparticles in food include oxidative stress reactions, protein denaturation and DNA damage. Around the world, there is still no consistent safety review methodology for nanoparticles, particularly for nano-based food. It is critical to regulating the use of nanoparticles in food by developing risk assessments and safety risk management techniques for nanoparticles in foodstuff that comply with 21st-century toxicity test aims and objectives.

## Declarations

### Author contribution statement

All authors have contributed significantly to the development and the writing of this article.

### Funding statement

This research did not receive any specific grant from funding agencies in the public, commercial, or not-for-profit sectors.

### Data availability statement

There are no data associated with this article.

### Declaration of interest’s statement

The authors declare no conflict of interest.

### Additional information

No additional information is available for this paper.
